# An exploration of patient-provider dynamics and childbirth experiences in rural and urban Peru: a qualitative study

**DOI:** 10.1186/s12884-021-03586-y

**Published:** 2021-02-15

**Authors:** Brianna Vargas, Paola Louzado-Feliciano, Nicole Santos, Shannon Fuller, Sopiko Jimsheleishvili, Ángela Quiñones, Holly H. Martin

**Affiliations:** 1 University of California San Francisco, Institute for Global Health Sciences, San Francisco, CA USA; 2 Department of Public Health Sciences, University of Miami, Miller School of Medicine, Miami, FL USA; 3 University of Miami, Miller School of Medicine, Miami, FL USA; 4 Professional School of Human Medicine, Catholic University of Santa Maria, Arequipa, Peru; 5 School of Medicine, University of California San Francisco, San Francisco, CA USA

**Keywords:** Childbirth, Labor and delivery, Maternal health, Health care delivery, Patient advocacy, South America

## Abstract

**Background:**

Between 2006 and 2013, Peru implemented national programs which drastically decreased rates of maternal and neonatal mortality. However, since 2013, maternal and neonatal mortality in Peru have increased. Additionally, discrimination, abuse, and violence against women persists globally and impacts birthing experiences and mental health. This qualitative study sought to better understand the attitudes and beliefs regarding childbirth among women and providers in Southern Peru. This study also explores how these beliefs influence utilization of skilled care, patient-provider dynamics, and childbirth experiences and identifies factors that impact providers’ provision of care.

**Methods:**

Thirty semi-structured interviews were conducted with 15 participants from rural Colca Canyon and 15 participants from urban Arequipa between April and May 2018. In each region, 10 women who had experienced recent births and five providers were interviewed. Provider participants predominantly identified as female and were mostly midwives. All interviews were conducted, transcribed, and coded in Spanish. A framework analysis was followed, and data were charted into two separate thematic frameworks using contextual and evaluative categories of conceptualization of childbirth.

**Results:**

All recent births discussed were facility-based births. Four domains emerged: women’s current birth experiences, provision of childbirth care, beliefs about childbirth among women and providers, and future health-seeking behavior. Findings suggest that women’s feelings of helplessness and frustration were exacerbated by their unmet desire for respectful maternity care and patient advocacy or companionship. Providers attributed strain to perceived patient characteristics and insufficient support, including resources and staff.

**Conclusions:**

Our findings suggest current childbirth experiences placed strain on the patient-provider dynamic and influenced women’s attitudes and beliefs about future experiences. Currently, the technical quality of safe childbirth is the main driver of skilled birth attendance and facility-based births for women regardless of negative experiences. However, lack of respectful maternity care has been shown to have major long-term implications for women and subsequently, their children. This is one of the first studies to describe the nuances of patient-provider relationships and women’s childbirth experiences in rural and urban Peru.

**Supplementary Information:**

The online version contains supplementary material available at 10.1186/s12884-021-03586-y.

## Background

In 2004, the World Health Organization (WHO) defined skilled birth attendants (SBA) as accredited health professionals who are appropriately trained to safely detect, manage, and refer complications during facility-based births and home-based births [1]. Data from multiple studies shows skilled birth attendance provided by a doctor, nurse, or midwife during childbirth decreases maternal and neonatal mortality [2–4]. This data includes a controlled trial in Pakistan which found a 30% reduction in neonatal mortality for the intervention group receiving antenatal, intrapartum, and postnatal care provided by SBA [4]. Furthermore, a systematic literature review including 60 Asian, African, and Latin American/Caribbean studies related to safe delivery (defined as hygiene, thermal care, and clean cord care) revealed a 44% increase of safe delivery in births attended by trained traditional birth attendants (TBA) compared to those attended by untrained TBA [3]. Despite this evidence that skilled birth attendance can improve birth outcomes for mothers and infants, approximately 45 million women globally delivered without skilled birth attendance in 2011 [5]. This is one of a larger pattern in which discrimination, abuse, and violence against women globally continues to negatively impact physical and mental health [6]. The provision or deprivation of respectful maternity care, the universal human right every woman has to the highest attainable standard of dignified and respectful reproductive health care impacts women, their mental health, and their childbirth experiences [7–9].

Between 1996 and 2006, Perú experienced significant decreases in maternal and neonatal mortality, moving from 99 to 68 maternal deaths per 100,000 live births and 11 to 8 neonatal deaths per 1000 live births [10, 11]. There have also been steady increases in antenatal care and SBA utilization, which can be attributed to various national efforts. These efforts include Proyecto 2000, which increased facility-based births access among newly insured women [12, 13]. Despite these improvements in mitigating birth-related mortality and increasing health care access, urban-rural disparities persist. A 2016 WHO report revealed that 98.9% of urban women and just 88.9% of rural women in Peru attended at least one antenatal visit with SBA, while 97.3% of urban women and only 71.3% of rural women gave birth with SBA [14]. Moreover, the decrease in birth-related mortality reversed. Between 2015 and 2017 maternal mortality rose to 88 maternal deaths per 100,000 live births and between 2012 and 2013 neonatal mortality rose to 12 neonatal deaths per 1000 live births [14, 15]. Maternal and neonatal disorders, including infection, asphyxia, prematurity, and pregnancy-related issues, still account for over one-third of total deaths among Peruvian children under-five [16, 17]. Additionally, rural and remote areas in the northern jungle regions and southern high-altitude regions, such as Colca Canyon, have a comparably higher burden of neonatal mortality when compared to large, urban cities such as Lima [10, 18].

Evidence regarding the factors that influence disparities among current childbirth practices and quality of maternal care in Peru is limited. However, in similar settings, factors that affect care access and utilization include: distance to care, income, education level, access to culturally appropriate services, respectful maternity care, patient-provider dynamics, health expenditure, and coverage of related childbirth interventions [2, 7, 8, 19, 20]. Factors that influence the quality of facility-based births include sufficient training for SBA, adequate equipment and facility staffing, functional team dynamics, and larger systems-level factors like funding and referral policies [1, 21].

Thus, this study attempted to: 1) better understand the attitudes and beliefs regarding childbirth among both birthing people and healthcare providers, 2) describe how those attitudes and beliefs influence skilled birth attendance, patient-provider dynamics, and childbirth experiences, and 3) identify factors that influence providers’ provision of care. All skilled birth attendants interviewed in this study are referred to as providers, encompassing the diverse group of interviewees including midwives, nurses, and doctors. All participants who experienced recent childbirth are referred to as women, because all of these individuals self-identified as women.

## Methods

Qualitative interviews were conducted between May and August 2018 to identify and better understand women’s attitudes and beliefs about childbirth. Additionally, we identified facilitators and barriers for the provision of medical care during childbirth from providers from both regions and providers attitudes and beliefs to explore current patient-provider dynamics. Women participating in the study also completed a basic questionnaire prior to their interview; these responses allowed us to describe the baseline characteristics and birth practices of the sample.

### Setting

**Fig. 1:**
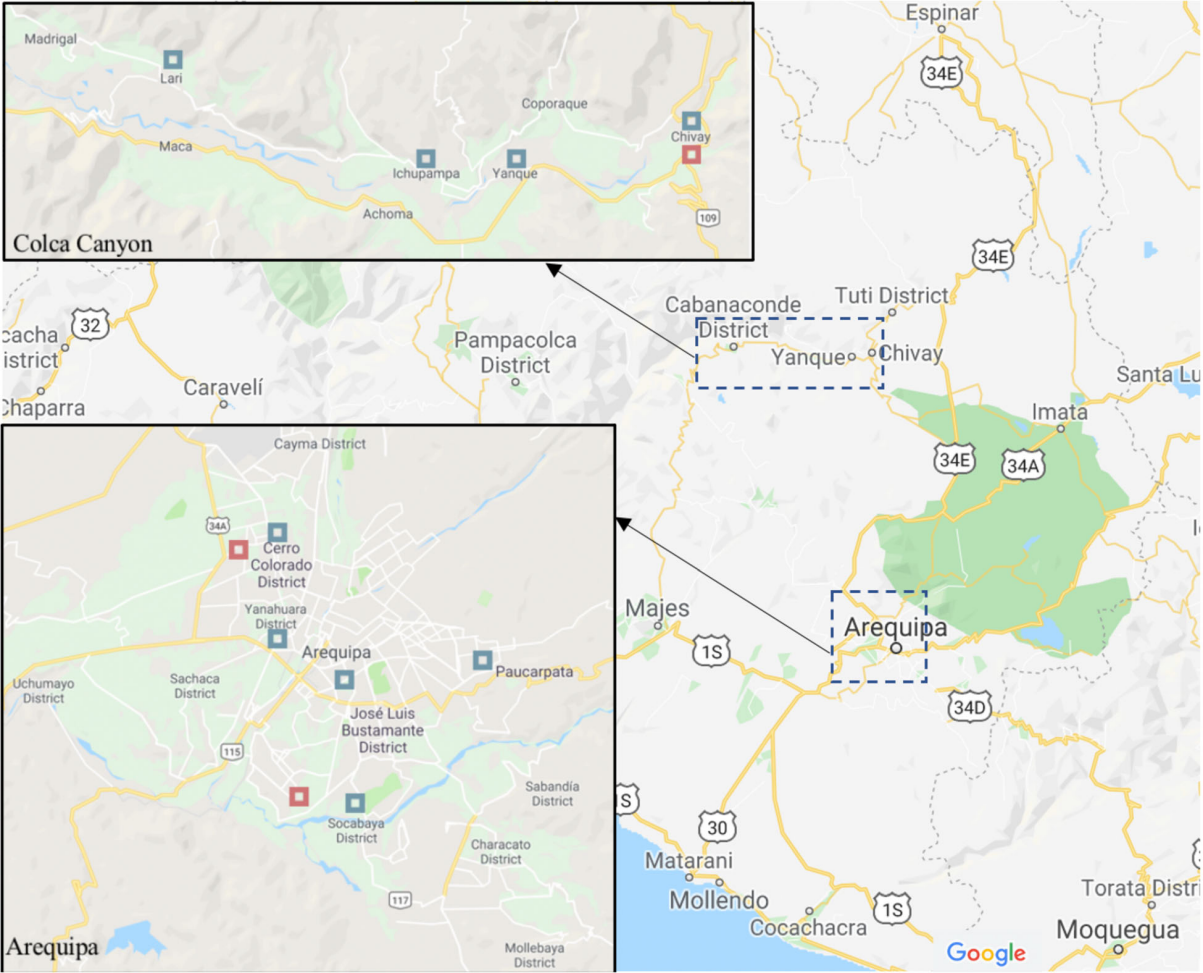
Google Maps of rural and urban recruitment areas for women and providers. Marks the recruitment areas for women and providers in Fig. 1. Images legally taken from Google Maps

Qualitative interviews were conducted in a city and a rural valley in two neighboring provinces of Southern Peru. The Arequipa (urban) and Caylloma (rural) provinces are roughly the same size geographically. Colca Canyon Valley, in the Caylloma province, is approximately 244 km (km) northwest of the city of Arequipa and is one of the closest and more densely populated rural areas relative to Arequipa. There are small *Ministerio de Salud* (Ministry of Health, or ‘MINSA’) health facilities classified as basic “level 1, category I-1” *puestos de salud*, health posts in the villages along Colca Canyon which offer basic health education, appointments, and referrals from trained community health workers or midwives. The only “level 1, category I-4” MINSA clinic that is equipped and staffed for facility-based births is in Chivay, up to 45 km from many rural villages. Additionally, the nearest “level 2, category II-1” MINSA facility equipped to provide emergency obstetric and newborn care for high-risk births is in Arequipa, 243.8 km from Chivay [22]. Provider participants were recruited from each of these three MINSA clinics across our two study areas. Women participantes were recruited from rural villages and urban districts throughout each of the two provinces (Fig. 1).

### Selection of participants

#### Women participants

Women who experienced birth between May 2016 and May 2018 were purposively sampled with the help of community leaders and organizations that work closely with mothers in Arequipa and throughout the Colca Canyon villages [23]. *Programa del Vaso de Leche,* a Ministry of Development and Social Inclusion program that strives to improve nutrition for young children was another collaborative partner [24]. This program’s group meetings across Arequipa and Colca Canyon were attended by a lead researcher to present the project and recruit interested women. The project was presented to over 60 women across both regions. Women were also recruited via snowball sampling, in which women who met our criteria were invited to the study by other community partners like clinic leadership or by women who were already interviewed [25]. These women were contacted directly to set up times and locations for the interviews. The 20 women that accepted were adults of reproductive age between 19 and 41 years old who delivered a baby between June 2016 and June 2018. This time frame ensures their experiences reflected current practices. All women participants were required to speak either Spanish or Quechua, and all chose Spanish as their preferred language.

#### Provider participants

Government authorization was obtained to recruit provider participants from three specific MINSA health clinics in Colca Canyon and Arequipa. A purposive sampling approach was used to recruit providers from each region. Leaders at each of the three approved MINSA clinics were contacted, after which project information was shared with their teams of doctors, nurses, and midwives. These childbirth care teams were larger in the urban settings and smaller in the rural setting. Lead researchers traveled to the three clinics and met with those who agreed to participate to present the participation and consent information. All 10 provider participants were currently practicing and had at least two years of childbirth attendance experience at the time of recruitment to ensure that they all had a baseline of experience working directly with women during labor, delivery, and post-delivery care. Provider participants were required to speaker either Spanish or Quechua, and all selected Spanish as their preferred language.

### Data collection

Data collection instruments were generated by bilingual principal investigators first in English and then in Spanish. Instruments were reviewed by our research partners in Peru for culturally appropriate phrasing and terminology. Study information and consent for study participation were reviewed with all recruited individuals. Verbal consent was obtained from all participants, as outlined in our institutions’ ethics approvals, and a copy of the consent form was distributed.

#### Demographic and childbirth history questionnaire

Women participants who gave consent to participate completed a one-time paper-based demographic questionnaire in their preferred language. The questionnaire was proctored by the interviewer in a quiet private space. If participants did not feel comfortable completing it independently, the interviewer guided them through the form verbally. The questionnaire included 22 questions regarding personal history of pregnancy, labor and delivery, and sociocultural characteristics (Supplemental File 1). The questions reflected the following variables: number of births with skilled birth attendance, number of facility-based births, number of home-based births, and clinical outcomes for women and their newborns. Clinical outcomes included self-reported complications, stillbirths, and neonatal deaths.

#### Interviews

All participants (both women and providers) who gave verbal consent to participate also completed an in-person 20–35-min semi-structured elicitation interview in Spanish. This interview technique is used to accurately probe sensitive, personal subjects; it allowed us to introduce our subject matter and ask non-leading or judgmental follow-up questions to interrogate the root feelings and understanding of events as the subject saw them [26]. Provider interviews typically took place during work breaks or before or after work shifts, while interviews with women participants were typically conducted before or after community engagements or appointments. Each interview took place in a private and quiet space, including personal homes and community spaces, and was conducted by a member of the research team who is a native Spanish speaker and trained in semi-structured interviews. Given the personal and sometimes emotionally evocative nature of the questions, participants were given ample time for breaks if needed. Though interviews for women and providers followed different guides, as outlined in the following two sections, all interviews included open-ended questions with additional probes exploring individual experiences. The interviews were audio recorded for transcription and translation, and the research team debriefed after each interview.

##### Women

Interview domains for women covered experiences pertaining to each of their childbirths, including childbirth preparation, birth attendance, additional support and resources, respectful and appropriate care, post-birth experiences, and community norms (Supplemental File 2). Interviews also explored the various factors in the women’s lives that influences skilled birth attendance. Each of the 18 questions included specific probes to further engage informants in the discussion of their attitudes and beliefs regarding labor and delivery. Women were prompted to walk the interviewer through each of their pregnancy and childbirth experiences in order from first to last. We gathered detailed narratives of their personal experiences with childbirth care and the nature of interactions with providers and other individuals present if applicable; this included anecdotes from before, during, and immediately after childbirth.

##### Providers

Interview domains for providers included patient rapport, facilitators and barriers to care, delivery of appropriate and respectful care, other methods of care, and post-birth practices (Supplemental File 3). The 15 questions contained additional probes to acquire detailed anecdotal information regarding their attitudes and beliefs around experiences with care provision across various settings and throughout their careers. The interview was also designed to gather information about provider experiences and feelings regarding both individual and systemic-level facilitators and barriers to patient rapport, care, and work responsibilities. Further questions probed how those large-scale facilitators and barriers impact provision of care.

### Data analysis

Quantitative data were analyzed using SPSS Version 26.0 to provide descriptive statistics while qualitative data were coded by two researchers with Dedoose Version 8.0.35. A framework analysis was followed and data was sorted according to key domains and themes [27]. Earliest analysis included familiarization through the review of interview notes and transcripts: reading, reexamining, and creating brief transcript memos summarizing key recurring and novel ideas that emerged. The research team created two codebooks, or thematic frameworks, one for women interviews and one for provider interviews. Each codebook highlighted recurring topics through codes and sub-codes that informed the research aims, namely, to understand participant experiences, attitudes, beliefs, benefits, and challenges in regard to current childbirth practices, care, and birth outcomes. The framework spreadsheet matrices summarized key information and included direct patient quotes from each interview. Themes regarding experiences, facilitators, barriers, attitudes, and beliefs that underlie skilled birth attendance and birth outcomes were identified and refined using constant comparison through transcript memos and framework spreadsheets. Themes were written in English and Spanish in order to ensure agreement on accurate translation from all researchers including English-only speakers, Spanish-only speakers, and bilingual researchers. Findings including direct quotes were translated to English by bilingual research team. All direct quotes were reviewed to ensure participant anonymity, and we removed all identifying data, including names and locations, before including findings and quotes in the framework matrices.

## Results

### Sample description

#### Women

A total of 20 women completed a questionnaire and were interviewed. This participant sample had a group mean age of 29.2 ± 7.53 years; 80% completed high school and 95% had a monthly income below 1710 soles (US$512). 55% of interviewees reported their most recent birth occurred within the last year and a total of 44 live births were reported among all respondents. Forty-two births were facility-based births and two were home-based births; the two home births did not occur between June 2016 and June 2018. All participants from Colca Canyon reported facility-based births either in Chivay, the only birth facility within a 150 km radius from their homes, or in Arequipa, the only birth facility within a 250 km radius from their homes equipped to perform emergency obstetric and newborn care for high-risk births. 30% of women participants reported having their recent birth in a rural clinic, while 60% were referred to Arequipa for their birth. (Table 1).

**Table 1:** Socio-demographic and live births characteristics among women participating in interviews by geographic location, May–June 2018 (*N* = 20)

Socio-Demographic and Live Birth Characteristics	Sample N N(%)	Colca Canyon n(%)	Arequipa n(%)
**Total Sample**	20 (100.0)	10 (50.0)	10 (50.0)
**Age**			
18–29 years old	11 (55.0)	7 (70.0)	4 (40.0)
30–39 years old	7 (35.0)	3 (30.0)	4 (40.0)
40 and older	2 (10.0)	0 (0.0)	2 (20.0)
**Educational Attainment**			
Elementary School	1 (5.0)	1 (10.0)	0 (0.0)
Middle School	2 (10.0)	0 (0.0)	2 (20.0)
High School	16 (80.0)	9 (90.0)	7 (70.0)
University	1 (5.0)	0 (0.0)	1 (10.0)
**Monthly Income (Soles)**			
200–600 soles	9 (45.0)	6 (60.0)	3 (30.0)
601–1000 soles	7 (35.0)	4 (40.0)	3 (30.0)
1001–1700 soles	3 (15.0)	0 (0.0)	3 (30.0)
1701 soles or more	1 (10.0)	0 (0.0)	1 (10.0)
**Occupation**			
Housewife	16 (80.0)	6 (60.0)	10 (100.0)
Student	1 (5.0)	1 (10.0)	0 (0.0)
Other	3 (15.0)	3 (30.0)	0 (0.0)
**Most Recent Birth**			
< 1 year ago	11 (55.0)	3 (30.0)	8 (80.0)
> 1 year ago	9 (45.0)	7 (70.0)	2 (20.0)
**Parity (total number of births)**^a^			
1–2 births	14 (70.0)	7 (70)	7 (70)
3–4 births	5 (25.0)	3 (30)	2 (20)
5 or more	1 (5.0)	0 (0)	1 (10)
**Distance to Nearest Birth Facility (minutes of travel)**			
< 30 min	14 (70.0)	8 (80.0)	6 (60.0)
> 30 min	6 (30.0)	2 (20.0)	4 (40.0)
**Parity by Facility Type**^a^			
**Total Sample**	44 (100.0)	21 (100.0)	23 (100.0)
MINSA Clinics	12 (27.0)	8 (38.0)	4 (17.0)
Government Hospital	29 (66.0)	13 (62.0)	16 (70.0)
Private Hospital	1 (2.0)	0 (0.0)	1 (4.0)
Home	2 (5.0)	0 (0.0)	2 (9.0)

^a^Total number of live births reported differ from total number of participants interviewed

#### Providers

A total of 10 providers from three MINSA clinics in Arequipa and Colca Canyon were interviewed. Among our respondents, 80% were female with a mean age of 52.6 ± 7.8 years. Participants had a mean work experience of 21.0 ± 6.4 years; 60% were midwives, 30% were doctors, and 10% were nurses. Five were recruited from Colca Canyon and five were recruited from Arequipa.

#### Themes

Themes from interviews with women and providers fell within the context of four key domains; six major themes emerged for women and four major themes emerged for providers (Table 2). Women living in rural and urban settings shared similar experiences; however, rural women had to transport themselves to Arequipa for “high-risk” births and discussed a feeling of being ‘othered’ in urban clinics and hospitals. Providers practicing in rural and urban settings shared similar experiences and beliefs in regard to patient-provider dynamics, though there were some distinctions regarding the facilitators and barriers experienced in each of the two settings.

**Table 2:** Domains and themes among interviews with women and providers, May–June 2018

	INTERVIEW THEMES	
**DOMAINS**	**Women**	**Providers**
**Women’s current experiences during facility-based births**	Patient advocacy & companionship	NA^a^
	Respectful maternity care	NA^a^
**Provision of childbirth care**	NA^a^	Facilitators that improved provision of care
	NA^a^	Barriers that interfered with provision of care
	NA^a^	Justification of disrespectful care
**Beliefs about childbirth among women and providers**	Norms within childbirth care expected among mothers	Perceived difficulties with patients expected among providers
	Patient blame	NA^a^
**Future health-seeking behavior**	Plans for future safe childbirth	NA^a^
	Resilience of mothers	NA^a^

^a^NA means not applicable

#### Domain: Women’s current experiences during facility-based births

##### Theme: patient advocacy & companionship (women)

This theme encompassed women’s experiences of advocacy for their needs and the needs of their baby from hospitalization to discharge. Women who experienced patient advocacy were either advocated for by a partner or close family member, or they self-advocated. A young woman who gave birth in Arequipa shared that her mother advocated for her and her child after she was taken to recovery with no update on the status of her child. Most urban women, however, reported a lack of birth advocacy and companionship during childbirth. This was a major difference between experiences in rural versus urban areas. Women who gave birth in less crowded rural clinics were often allowed a companion and reported feeling supported and safe; one rural woman who gave birth in a rural clinic said, “I didn’t have anything to feel scared about with my husband there with me through the pains.” Typically, laboring women in urban facilities were directed into a room—a room which often already contained one or more women also in labor—while accompanying family members and partners were directed to waiting areas away from the birthing women. Many women expressed that they would have felt more comfortable with their companions nearby because they would have lent compassion, understanding, and most importantly moral support that they did not receive throughout childbirth.

“Well, I have talked with many people that gave birth in clinics where they said they let their family members come in, they can receive visits, even where the father can be with the mother so that he can give emotional support. Not with government insurance [facilities]. To me that is very bad because when I was alone, my sister wanted to accompany me because I was scared. The first-time moms always have that feeling but they did not let her. They made her wait and made me wait somewhere else. There was no one to help me, so the worst part is that they take me away from my family member that I feel safe with and they complicate the situation.” *–*mother, Colca Canyon

One woman specifically described a stark contrast in how providers treated women who did have companionship and those who did not:

“ … the treatment was not very good. Luckily, I had a family member that worked there [doctor] and tried to minimize that [negative treatment] because in reality, I am not going to lie, they treat everyone [badly]. And I do not like that. They see that we are pregnant, and they should be more careful during childbirth. Well, they helped me and then they would not treat me like that, but they were a bit more aggressive with the other patients.” *–*mother, Arequipa

In a few rare cases from both rural and urban areas, women experienced childbirth without their loved one but reported that their providers, often midwives, advocated for their needs and offered support. These spaces also facilitated self-advocacy as women said they felt more validated and much calmer.

##### Theme: respectful maternity care (women)

The importance of respectful maternity care was expressed across all interviews and there was not a clear difference between rural and urban areas. When women experienced respectful maternity care before, during, or after childbirth, they also reported a higher overall quality of their childbirth experience. Respectful maternity care was described as encouragement, genuine care, and clear communication by providers. Respectful maternity care was typically more prevalent in smaller urban and rural MINSA clinics and one private hospital in Arequipa. In these instances, providers were described as helpful, psychologically supportive, and understanding of women’s needs and experiences with pain and discomfort during childbirth.

“… the young doctor really talked to me. She asked me if I felt like I could give birth naturally, if I had strength. I told her yes, I was only scared about the old [cesarean] scar. But I decided to give birth naturally … they said if there is any complication, they would do the cesarean, but they told me they had more faith that it would be normal and that nothing would happen to the old scar … [I felt] a bit calmer and safer.” –mother, Colca Canyon.

When women from both regions experienced disrespectful maternity care, they often reported a much lower overall quality of their experience. Most often, this was the case for childbirths in busy national hospitals and larger MINSA clinics. An overwhelming majority of women recalled experiencing disrespectful care from one or more of the providers attending a recent childbirth. Collectively, women expressed that they experienced discrimination, neglect, verbal abuse, and sometimes physical abuse through the aggressive nature of interactions with providers. One woman described how the nurse ignored her pleas for pain medication after her cesarean and was aggressive when she finally performed the injection later on. Many women also compared maternity care from one birth to another.

“In the first [pregnancy]...they came and then would insert [their hand] and I would say it hurts and they would say deal with it, and they would insert [their hand] anyways. But in the second they would tell me, ‘I am only going to touch you only once, do not worry, you will continue to advance.” And they would try to motivate me, they would not touch me inappropriately a lot. But in the first one horrible, and every half hour, I think. –mother, Colca Canyon

In addition to verbal and physical abuse, disrespectful care in the form of neglect and discrimination was common among both groups of women and also led to negative perceptions of providers. One woman said that the midwife on night shift never checked on her or her infant throughout the 8 h following childbirth. Another who gave birth alone shared that the doctor and midwife both left the delivery room after checking the infant without saying anything to her. A woman in Arequipa reported feeling upset and confused when interns who could not answer her questions were the only ones to check on her.

“… I was upset because there were no doctors, because they would not tell me what happened to my baby and no one would tell me anything. They would just say he was in observation, but they would not even say ‘miss, if you want more information … ’ or my husband could have gone, I do not know. We [woman and husband] would ask, but only the interns would come, and they are different [from the doctors].” –mother, Arequipa

Women in both groups also sometimes felt that they were treated differently by providers because of their age, socio-economic status, level of education, or cultural traditions. Most women described that the behaviors and attitudes of the providers made them feel uneasy and upset.

“… Since I was young–I was 17–there were some nurses that were surprised … they would not yell but it made them uncomfortable that I was too young. That is how it felt, that is how I felt. I felt, like uncomfortable also because of the way they looked at me. I do not know, they would tell me things … but then like I said I did not feel regret or anything, but uncomfortable.” –mother, Arequipa

“Over here [Colca Canyon] traditionally, after the birth, we do not touch any water. But in Arequipa they force you and they do not understand you. You have to bathe even with cesarean and everything … so in that part they are not understanding.” –mother, Colca Canyon

#### Domain: provision of childbirth care

##### Theme: facilitators that improved provision of care (providers)

Providers outlined many factors that improved the ways in which they could provide efficient, safe childbirth. Two of these key factors described across both regions were large clinical spaces for increased mobility during childbirth and a large team of support staff. A majority of providers said the availability of a cohesive team of providers including doctor, nurses, midwives, and midwife interns in the facility helped them remain calm because they could call on a larger team to lend support when a complication arose, or a patient was in distress.

Many rural providers expressed that working in a facility serving a small population and lighter workload can be an important facilitator of efficient childbirth care and lends itself to fewer patients and births. Comparatively, although providers in smaller urban facilities have much higher workloads often attending three or more births at a time, they described that they had more opportunity to build trust with women than busy national hospitals.

“Many times, we have had patients tell us that the care we give them here [smaller urban MINSA clinic] is better than those who attend births in [national public] hospital. Here there is more trust, they have seen their providers many times. They even know them by name, while at the hospital they don’t know anyone, and the care has to be rushed. In that sense there have been a lot of patients that prefer being here and not a hospital. And well sometimes we have patients whose births get complicated and they absolutely refuse to go to a hospital.” –Midwife, Arequipa

Providers practicing in rural settings were able to build a deeper relationship with patients because of their increased interactions with women both inside and outside of the facility compared to urban settings.

“And I know them [patients] because I have lived here. It gives me that confidence, I feel comfortable going to them. Like when I am not working, I am just another person in the village. I go around, I go to the plaza and I talk to the women, I laugh a bit, and everything is nice.” –Midwife, Colca Canyon

##### Theme: barriers that interfered with provision of care (providers)

Regardless of the location in which they practiced, providers noted various factors that interfered with the quality of safe childbirth and respectful maternity care they wanted to provide. A majority described an environment in which there are currently more barriers than facilitators. One of the biggest barriers across both regions is the need to refer women to more equipped and consequently over-populated national hospitals due to a lack of both equipment and personnel. Providers from both rural and urban clinics reported lacking ultrasounds, specialists like pediatricians, and operating rooms. One doctor in Colca Canyon shared his frustration in having to refer women to Arequipa, 250 km away, to have cesareans because his clinic does not have an operating room or supplies to treat emergency complications. Another doctor in Arequipa shared that the lack of equipment at hand to make quick and easy diagnoses is a barrier to care and could lead to more serious problems for the mother or infant.

“The truth is that the infrastructure is not adequate. I would like to have equipment here in the room that would allow me to give a more accurate diagnostic and that limits me. At my previous job we used ultrasound equipment and so I could determine any disease or risk factor with more certainty and address it directly. Instead, when I give that task to another provider … sometimes it does not coincide, and the margin of error is large and there could be a more serious complication.” –Physician, Arequipa

One barrier discussed by doctors in the Arequipa clinic was the lack of additional doctors on call. The team of nurses and midwives is extensive, with as many as eight midwives on call on the same shift, but there is only one gynecologist on call per shift who has to attend all obstetric appointments, family planning, live births, and emergencies in busy urban clinics on their own. The doctors expressed that this situation led to limited face to face time with patients, which also interfered with patient-provider communication and the quality of their relationships with patients.

##### Theme: justification of disrespectful care (providers)

Only providers in Arequipa stated that the limited time available to them throughout their days is a major reason for women feeling unsupported. They said that instances of neglect were defensible because of the high volume of births that occur and their need to attend all of their patients.

“… there are few midwives here and sometimes there are many births at the same time. So sometimes there is time [to attend and establish support] but other times there is not. That is a weakness because some of the patients sometimes feel alone. They do not feel that accompaniment by us [providers].” –Midwife, Arequipa

Many providers talked about instances in which they had negative reactions toward patients during childbirth because of patient characteristics, behaviors, or expressions of strong emotions. Negative reactions included providers raising their voices toward patients, getting visibly frustrated, and reprimanding or scolding patients when they were uncooperative. Most providers across both settings justified these actions by stating that they needed to do what was necessary for the safety of the child. They shared that sometimes women would thank them for their commitment to the child, but other times it made women visibly unhappy. Regardless, providers felt that their actions were effective because women would finally cooperate and ultimately the childbirths moved along successfully. A midwife shared that her own personal experience in having a complicated childbirth and a disabled child as a result also influences the way she interacts with patients.

“They are not always happy, one here or there because we scold them and sometimes the explanation is not there, but it is because we get desperate that they are not pushing correctly or sometimes they drink their [traditional] teas … They do not push how they are supposed to, they do not listen. The women worry more about their pain and not their baby and I give them attitude because my older daughter is [disabled] because of problems with childbirth and cesarean … So with my prior experiences, I make sure that the baby does not suffer and comes out healthy.” –Midwife, Colca Canyon

#### Domain: beliefs about childbirth among women and providers

##### Theme: norms within childbirth care expected among mothers (women)

Women discussed a shared belief that negative experiences and poor treatment during childbirth are commonplace and “to be expected.” One mother said that she prays and hopes for the best because it is luck whether you get “good or bad doctors.” A common theme that arose among women who reported overall positive childbirth experiences was that they all believed they got lucky with the providers that attended their birth because it is a common experience among many other women in their community to have negative childbirth experiences. A large majority of women also revealed that they witnessed the negative treatment of other women in birthing facilities or constantly heard about negative treatment among their social network of mothers.

“It was the nurse’s job [to support], and yes she treated all of us poorly. I saw the others [women] that came in and we all felt like that.” –mother, Arequipa

“… we know they do not care about others pain; it is like they are rocks. They do not feel anything, and they are indifferent, that is what I felt.” –mother, Colca Canyon

One woman described that even after paying a form of private insurance, which she believed would improve her quality of care, she still experienced neglect from providers. She also shared that other women do not want to go out of their way to purchase health insurance if treatment will be the same or worse.

“If we pay, we need the same attention as everyone else, not that they put us aside because we do not belong because it is a government insurance facility … Sometimes the women I gave birth with would say, ‘on the contrary [treatment] should be better because we are paying.” –mother, Colca Canyon

Second or third-time mothers who gave birth in busier urban facilities reported that the shared expectation of negative treatment during facility-based births led them to form an alliance with other mothers in childbirth to create a safe space where they could lend each other the support that was lacking. Many described that they would try to encourage and help one another from the time of hospitalization to discharge. One woman said that after giving birth to her third child, she had to help the new mother in the neighboring bed learn how to breastfeed, as the providers would not come offer support to her or the other women.

##### Theme: patient blame (women)

Women shared that family members, community members, and providers projected blame onto them after negative childbirth experiences. These individuals told women that they were responsible for their own mistreatment due to their behaviors or getting pregnant again after negative facility-based births experiences. Many women internalized this blame and felt shame. One woman noted that her family and friends advised her to stop having children “to save herself the trouble.” Another said that her providers rushed her into an emergency cesarean and then her close family were upset with her for “choosing a cesarean.” Many women also revealed that providers often projected blame regarding caring for their infant during recovery.

“I would ask [about the baby] and no one knew. [I would say,] ‘Miss, what is going on? They will not bring him.’ And they would just tell me that they would ask … And since I was still bad, I tried to go but halfway down the hall I could not walk anymore and so I had to go back because I did not even know how far [the baby] was. Another whole day passed, and I was able to walk and when I went to the neonatal area [the nurses yelled] ‘Miss, what happened? You are supposed to bring diapers for your child and come to feed him!’ But no one told me! I am not a psychic and I asked [providers about the status of the child]. It is not like I was just thrown on the bed.” –mother, Arequipa

“They told me I had to do it [cooperate] for the baby. If not, if I didn't do my part, the baby was going to die, or he could be traumatized. So that was a real difficulty for me [pause] … I don't know psychologically. They worked me psychologically.” -mother, Colca Canyon

##### Theme: perceived difficulties with patients expected among providers (providers)

In addition to previously noted interpersonal and system-level barriers to care, providers described that difficulties such as patient behaviors or perceived patient characteristics were particularly troublesome as they interfered with completing a safe childbirth and affected patient interaction and treatment. For example, providers stated that women who came with their own Andean or indigenous cultural birthing traditions interfered with their ability to provide effective care as medical providers. One rural midwife shared an experience in which the patient drank the *zapallo* root, a traditional tea that increases uterine contractions and thus speeds up labor. She described this as extremely frustrating because the team of providers was not ready for the delivery and had to rush her to the delivery room; she recalled wondering what terrible things could have occurred if they had not gotten her into the delivery room in time.

Across both regions, providers also described patient characteristics or behaviors that led them to believe that a woman was unprepared or indifferent about her childbirth or child. These included education level, prenatal class or scheduled check-up attendance rate, socio-economic status, and hometown. Providers often drew conclusions about these women and felt that as providers, they needed to work harder to complete a safe childbirth. One urban provider described that patients with “more education” were easier to work with because they knew what they were doing, while those with “less education” were more difficult. Rural providers shared that their patients would come into the facility with dirty or old clothes for their infant or did not want to cooperate with providers during labor and delivery because of their traditions or lack of knowledge.

“… it is important to educate the pregnant mothers because sometimes they come from other zones, they come from Puno, Cusco, they come with their beliefs and traditions. So, we are missing the education. Sometimes nowadays, they get there, and we do not have time to orient them or do their prenatal check-ups.” –Midwife, Arequipa

These difficulties also included patient behaviors. At times, providers across both regions felt that patients were being difficult or exaggerative during childbirth by crying, complaining, or failing to push. One midwife explained a situation in which her patient, accompanied by the husband, was agitated and did not want to cooperate when she was being told to push.

“Then sometimes they don't push. I say, look I'm doing my job, we're all doing our job, if your wife doesn't push, if your baby is born bad, it's no longer my responsibility, it's no longer the doctor's fault, it's nobody's fault, just hers and then he [the husband] says, ‘Push!’ … Other times the husband says, ‘Don't scold her, don’t yell at her.’ Then I tell him, run outside, I throw out the husband.” -Midwife, Colca Canyon

#### Domain: future health-seeking behavior

##### Theme: plans for future safe childbirth (women)

The two women who experienced home-based births over 13 years ago both said that they would have preferred to give birth in a facility. One recalls that after an accident she was forced to give birth at home without the presence of a provider because they were too far from help. Her baby suffered asphyxia and cerebral palsy. “I did not want to give birth like that,” she said as she cried. The other woman shared that she would always rather give birth in a facility even though her experience at home provided more comfort, safety, and support from her family.

“It scares me to give birth at home again, Miss because … What if she doesn’t come out, Miss? She could suffer asphyxia, or the membrane could rupture, and she could drown inside. It is more secure with the doctors even if they complain.” –mother, Arequipa

Regardless of negative experiences, each woman stated that they chose to give birth in a facility setting because of the safety associated with provider knowledge and expertise and facility resources in case of any emergency. Though their negative individual and community experiences contribute to women not wanting to go into facilities, the safety of their child remained the driving factor that determined their ultimate decision to give birth in a facility.

“The treatment of the patient; for example, for me I just pray. I do not want to go to the hospital for many reasons: disease and there are so many things that you see over there that I do not want to go. Oh well, I go for my children. I have gone to the hospital for the three of them because here [rural village] the midwife sends you to Chivay or Arequipa depending on your state for the safety of the baby.” –mother, Colca Canyon

##### Theme: resilience of mothers (women)

Despite the lack of respectful maternity care and patient advocacy during many women’s childbirth experiences and patient blame by childbirth staff or community members, women exhibited resilience in overcoming stressful and traumatic moments during childbirth.

“I was traumatized because they would just yell, ‘push, push!’ and I did not know how to push and oh! It traumatized me more … the first time yes, it made me want to say no more children! But, look, here we are [laughs, then pauses and looks down at her breastfeeding infant].” –mother, Colca Canyon

All women interviewed demonstrated resilience through their ability to overcome perceived difficult experiences and the associated negative emotions. One of the key pillars associated with resilience is adaptability in the face of hardship or risk and maintaining a strong sense of self care or self-soothing. Every single woman who reported a negative experience in an urban setting, also shared how they adapted on the spot or during their later births. Some examples were tuning out mentally, thinking about their family, or becoming passive as a means of self-preservation during challenging moments.

“This time [recent birth] I tried not to complain too much, I put up with the pain, endured, and I knew how to control myself. They [providers] gave me more support and would tell me what to do without yelling because sometimes you don’t control your pain and sometimes in that moment you don’t know what to do and you get desperate and that is when sometimes the midwife can grab you and tell you something, something disrespectful.” -mother, Arequipa

Many times, they also did this by shifting their attention to the needs of their infant or their older children. In spite of adversity and the fear of experiencing disrespectful care again, the women interviewed across both regions shared that they each became stronger advocates for themselves, their future children, and other women in their communities and families with each childbirth.

## Discussion

The discussion reflects the four domains: women’s current experiences, provision of care, beliefs about childbirth among women and providers, and future health-seeking behavior.

The fact that all participants’ most recent birthing experiences took place in facilities reflects the overwhelming shift in current experiences towards facility-based births in both urban and rural Peru. However, the increase in these births seems to come at the expense of the previous practices as described by our women participants. Foremost among these is companionship and advocacy in childbirth; previous practices included the presence of family and spouses, community birth coaches (doulas), or traditional healers who offered the type of companionship women desire. A meta-analysis reviewing 41 global studies conducted from 1989 to 2015 concluded that women appreciated support by a companion of choice at birth. They experienced this desired companionship as a buffer to low expectations and experiences of disrespectful care at facilities [28]. Women who had negative facility-based experiences emphasized the disrespectful care and lack of patient advocacy and companionship. This non-respectful maternity care typically exacerbated their feelings of helplessness, anxiety, and fear before, during, and after childbirth. These findings are consistent with those outlined in a literature review compiling data from many African, Latin-American and Caribbean countries [29]. Labor and delivery is a physically, mentally and emotionally demanding experience and can be more intimidating for young and first-time mothers. Disrespectful care during and after childbirth negatively influences women’s demeanor during childbirth, the patient-provider dynamic, and future choices regarding childbirth facilities and care, and is also widely reflected across various countries including Mexico [8, 29–31].

Women’s cooperation with providers often decreased when they felt unsupported, and this greatly impacted provision of care. Their lack of cooperation exacerbated providers’ feelings of dissatisfaction and frustration with patients they labeled as “exaggerative” or “difficult.” This pattern is also seen across studies in middle and low-income countries [21]. Moreover, the percentage of deliveries in these facilities has increased greatly over the last decades and is still increasing incrementally; clinical directors and physicians confirmed that provider workload has increased in parallel [32]. Consequently, as seen in countries with similar trends, providers in both regions shared that a major barrier to adequate provision of care is lack of staff capacity and larger teams [21]. Additionally, with the shift to facility-based births, previously utilized traditional home birth attendants or *parteras* were not incorporated into the health system. Instead, providers said, informal TBA practically disappeared from birth practices altogether. This reintegration has the potential to improve mother-centered childbirth care and change provision of facility care, similar to the effect of integrated doulas or other birth coaches in other countries [33].

There was a shared community belief among childbearing women that patient blame and negative facility experiences were commonplace. The women in our study experienced patient blame from providers, family, and community members and were told that if women know experiences will be negative, they should simply stop having children. Women across qualitative studies in Mexico and rural Peru described similar experiences of patient blame, and a USAID landscape analysis comprising 18 countries indicated a normalization of this disrespect, abuse, and blame [2, 29, 31]. There was a common community notion among providers that women were often difficult to work with and did not have their child’s best interest at heart. The perception of patient difficulty seemed to be brought on by implicit biases roused by indicators of women’s status, social class, or background. This phenomenon has been studied across many other Latin American countries, and often includes bias against indigeneity [31]. It is critical to note that providers in our samples found it much easier to work with women who they perceived as being well prepared for birth; they associated perceived preparedness with higher status, education, and practicing ‘acceptable’ or ‘proper’ behavior during childbirth. These biases impacted the childbirth experiences of the women interviewed in our study, who predominantly self-identified as housewives, had unplanned recent pregnancies, and shared that they felt underprepared for childbirth. 95% of our women participants lived below the national monthly living family wage of 1710 soles (US$512) [34]. This indicates that in regard to social class, a majority of the women participants are classified as low-income; the lower range for middle-class is a monthly income of 1942 soles (US$584) [35]. Additionally, rural women with high-risk pregnancies are referred to Arequipa due to the lack of specialists, facilities, and equipment at local clinics. Urban providers may not understand or appreciate the unique traditions, beliefs, and notions about health and childbirth held by these women, further contributing to patient-provider strain [2].

**Fig. 2:**
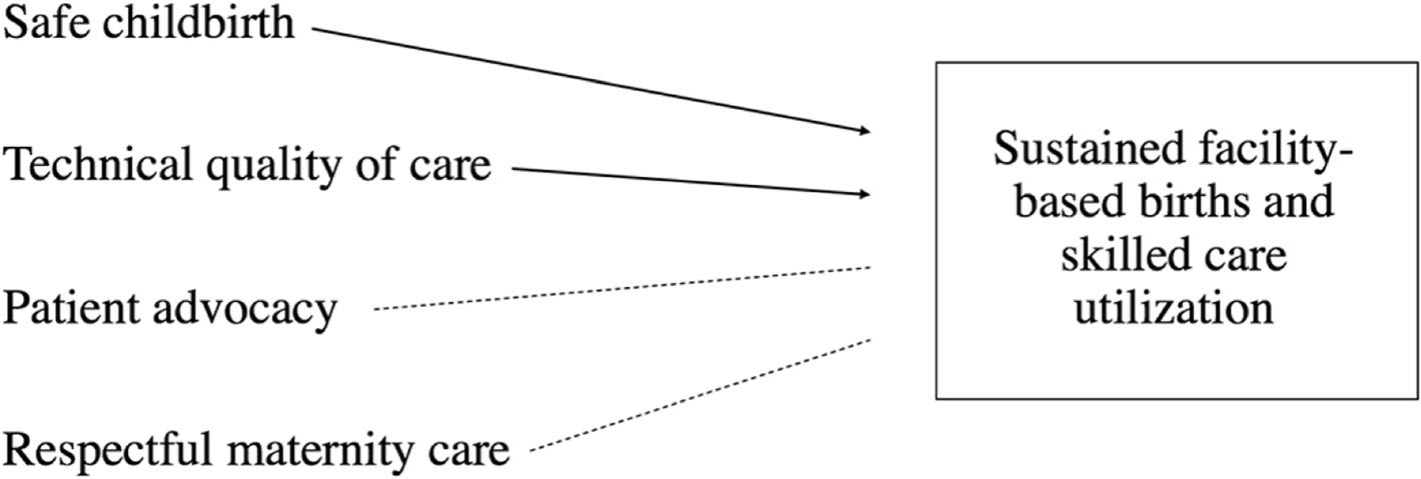
Women’s drivers and needs and pattern of care emerged through this analysis

**Fig. 3:**
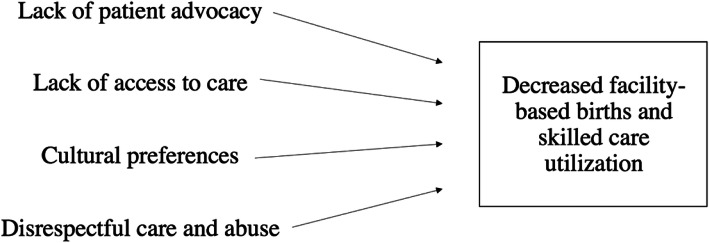
Pattern emerged among data from other countries and potential outcome of continued lack of respectful maternity care in Peru

The most important birth outcome to both women and providers was infant safety. Providers became dismissive of women’s needs and desires when perceived patient behaviors, characteristics, or traditional beliefs were seen as interfering with safe delivery [31]. From the opposite perspective, women expressed how respectful maternity care and safe childbirth spaces created by providers in lieu of a personal companion positively influenced the way they felt about providers and their desire for future births in facility settings. However, more often, the women in our study felt they had negative birthing experiences lacking in respectful care and patient advocacy. Regardless, for all women across both regions, safe childbirth was the main driver of decisions regarding skilled birth attendance in facilities. The women we interviewed chose to have facility-based births and stated that they believe they would continue to do so regardless of their own prior negative experiences or the community understanding that negative experiences occur in these facilities (Fig. 2). These findings lie in opposition to data from other low- and middle-income countries, which show that continuous disrespectful care (i.e. abuse and discriminatory actions) and negative experiences (i.e. patient blame) discourage skilled birth attendance and decrease facility-based births among women over time (Fig. 3) [2, 8, 29].

While these findings are important, this study is limited. Community leaders, who acted as cultural brokers, shared that many women they knew of who had experienced more severe negative childbirth experiences and outcomes, such as stillbirths, did not wish to be interviewed. These stories are therefore absent from this study. Additionally, though we conducted interviews until we reached thematic saturation, our samples were small and not representative of the large urban and rural regions we covered. Due to the specificity of our Peruvian ethics approval coming from MINSA, we only recruited providers from three authorized MINSA clinics in the two geographic settings. The five providers recruited from Colca Canyon were a representative sample of providers due to its smaller population and number of clinics, but the participants from Arequipa were not representative, as we recruited providers from only two of the existing 38 MINSA Arequipa clinics. We did not include providers working in non-MINSA private clinics or in the larger and public hospitals. Therefore, we can only draw conclusions about MINSA clinics.

## Conclusions

Our findings set a foundation for further research regarding culturally appropriate, respectful maternity care practices throughout Peru and beyond. Across rural and urban Peru, there is a disconnect between birthing people and providers, particularly around disrespectful maternity care. While future steps must focus on the patient-provider dynamic and respectful maternity care for all childbearing individuals, part of the solution might include eliminating provider barriers like workload and staffing. Furthermore, as the demand for childbirth care among pregnant people continues to increase parallel to the medicalization of childbirth in Peru and Latin America, we can anticipate a corresponding increase in disrespectful maternity care across facilities. These findings reflect a sense of increased care for infant safety and decreased care for the mental, physical, and emotional well-being of childbearing individuals and could be linked to the recent increase in maternal mortality during our period of fieldwork. Given the phenomenon across other regions described in Fig. 3, this increase in negative experiences for women could lead to a later decrease in utilization of skilled birth attendance in facilities. Further mixed methods research is needed in order to fully inform potential interventions or systems-level changes, but we hope these findings can begin conversations about how to move forward.

Neonatal and maternal mortality continues to increase. Over the last few decades continued improvements in equipment, provider training, and knowledge about safe childbirth have contributed to some positive outcomes; these advances should also be improving maternal and neonatal health, yet they are not. Given the implications of these findings and what we know from the emerging research on respectful maternity care and its impact on future health-seeking behavior and maternal health, we must continue to improve and expand upon the overall quality of childbirth care. All childbearing individuals, regardless of cultural background and socioeconomic characteristics, deserve the right to respectful maternity care, which goes far beyond the technical quality of safe childbirth.

## Supplementary Information

**Additional file 1: Supplementary File 1.** Women’s Questionnaire.

**Additional file 2: Supplementary File 2.** Women’s Provider’s Interview Guide.

**Additional file 3: Supplementary File 3.** Provider’s Interview Guide.

## Data Availability

Key data analyzed including quotes, tables, and figures in English are included in this published article. Data collection tools in English and Spanish are publicly available in the Open Science Framework data repository: https://osf.io/xfuen/. The two framework analysis tables (women and providers) contain potentially identifiable information that could compromise anonymity for our participants. These datasets can be made available upon reasonable request.

## Abbreviations

MINSA: Ministry of Health/Ministerio de Salud
SBA: Skilled birth attendants
TBA: Traditional birth attendants
WHO: World Health Organization
