# Supplementary material for: An exploration of patient-provider dynamics and childbirth experiences in rural and urban Peru: a qualitative study
Source: BMC Pregnancy Childbirth. 2021 Feb 15;21:135. doi: 10.1186/s12884-021-03586-y (PMC7885576; doi:10.1186/s12884-021-03586-y)
Supplement: Supplementary file 1 — Additional file 1: Supplementary File 1. Women’s Questionnaire. [file 12884_2021_3586_MOESM1_ESM.pdf]

ID #: _____________
Fecha: ____________
Entrevistador: ______

Questionnaire (22 questions)

(Please write a response on the line or select an option)

1. What is your age? ____________

2. What do you work on?

   ____

3. One-month household income:

   ____

4. What is date of your most recent birth?

   ____

5. What religion do you identify with?

   ____

6. What district/city do you live in?

   ____

7. Where were you born?

   ____

8. What is your highest level of studies?
   No school
   Primary school
   Middle school (grades 7-9)
   High school
   University

9. What is your marital status?
   Single
   Hunted
   Divorced
   Separate
   Widow

10. Distance to the clinic closest to your home?
    <30 minutes walking
    >30 minutes walking

11. Was your most recent pregnancy planned?
    Yes
    No

12. How many children do you have?
    1
    2
    3
    4+

13. How many times have you been pregnant?
    1
    2
    3
    4+

14. How many childbirths have you had?
    1
    2
    3
    4+

15. How many of your children died during childbirth?
    0
    1
    2
    3
    4+

16. How many of your children died within the first 28 days of life?
    0
    1
    2
    3
    4+

ID #: _____________
Fecha: ____________
Entrevistador: ______

17. How many times did you experience a complication during childbirth?
0
1
2
3
4+

18. If at least one complication arose, what was the complication(s)?

____

____

____

19. How many times have you given birth in a facility? Elaborate: _______________
0
1
2
3
4+

20. How many times have you given birth at home?
0
1
2
3
4+

21. How many times (at home) have you given birth with the help of a midwife, doctor, or nurse?
0
1
2
3
4+

22. How many times (in clinic) have you given birth with the help of midwife, doctor, or nurse?
0
1
2
3
4+
