# Supplementary material for: An exploration of patient-provider dynamics and childbirth experiences in rural and urban Peru: a qualitative study
Source: BMC Pregnancy Childbirth. 2021 Feb 15;21:135. doi: 10.1186/s12884-021-03586-y (PMC7885576; doi:10.1186/s12884-021-03586-y)
Supplement: Supplementary file 2 — Additional file 2: Supplementary File 2. Women’s Provider’s Interview Guide. [file 12884_2021_3586_MOESM2_ESM.pdf]

Interview Guide (Women)

Introduction:
"Thank you for taking the time to talk to me today. The purpose of this interview is to listen to women regarding their experiences with childbirth. There are no right or wrong answers to these questions, I would like to hear your honest and personal opinions on these topics. We look forward to using this learning to better understand these collective experiences and use these ideas to make suggestions for improvements in childbirth both at home and in the clinic. The interview is expected to take 45 minutes to an hour, depending on your answers. If you have any questions during our conversation, feel free to ask me. I would like to make it clear that you do not have to answer a question if the question makes you uncomfortable, and you can pause or stop the interview at any time. Our whole conversation will be recorded in audio, but everything we discuss will remain anonymous. Your name and information will not be connected to your answers.
If you are ready, can I start recording?"

Interview Questions:

1. First of all, I like to know how many children you have and how old are they?

2. Of your births, which was the most memorable and why?

"Let's talk about each of your birthing experiences, let's start with the first one."

Childbirth Preparation

3. How did you prepare for this birth?
   - Did someone help you prepare?
   - Why did you choose to prepare this way?

4. Did you give birth at home or in a clinic?
   - What are your opinions about giving birth at home or in a clinic?

5. What factors, if any, contributed to the way you prepared for this delivery?

Childbirth Assistance

6. Who was with you during this birth?
   - Who helped you the most and in what way?
   - Who did you want to be present?
   - Was anyone missing? Why were they absent?

7. Tell me about who cared for you during labor and delivery.
   - Were they family?
   - Were they doctors, nurses, midwives, or someone else?
   - How did they serve you?

Additional Support and Resources

8. During labor and delivery, did you ever feel you needed additional support or resources?
   - Did you have any complications?

- How did you know you were having complications?
- What kind of support or resources did you need?

9. If so, did you receive the support or resources needed? Why or why not?
   - What happened?
   - How did you take action?
   - Did anyone else take action for you?

10. Who participated in making decisions during this experience?
    - Were you the only person making decisions?
    - Did anyone else make a decision for you?
    - If so, who?
    - How did you feel?

<u>Respectful and Appropriate Care</u>

11. Did you feel respected or disrespected in this experience? Please tell me more about this experience.
    - Who made you feel this way?
    - What did they say or do to make you feel this way?
    - Did someone force you to do something you didn't feel comfortable with?
    - How did you cope with these feelings?

12. During this experience did you ever feel that the person caring for you did not understand your needs?
    - What was it about?
    - Who was involved?
    - Did they say or do anything specific to make you feel misunderstood?
    - What was the result?

13. Can you tell me about a time during this birth when you felt truly understood?
    - What made this experience better for you?
    - Who made this experience better for you?
    - What was the result?

<u>After Childbirth</u>

14. What happened immediately after your child was born?
    - Did those who attended him pay attention to the baby?
    - Who cared for you and who cared for the baby?
    - Who did you trust with your baby?

15. What was the result of this birth?
    - Did you or your child need emergency care? Please tell me more about this experience.
    - What happened to your child?
    - Did your child survive?

"Now let's talk about your second birth."
Repeat each domain

16. Tell me about a time when you were satisfied with your experience or birth result?

Community

17. What are some of the conversations you have had with women in your community about your birthing experiences?

-Can you speak with family, friends, neighbors?

-What do your family or friends think about your experiences?

18.Before we end our conversation, is there anything else you would like to add in regard to your experiences?

Conclusion:

"Thank you for taking the time to talk to me today, I appreciate your responses. Do you have any questions? If not, I'll turn off the recording now."

UCSF Institute for Global Health Sciences
