# Supplementary material for: An exploration of patient-provider dynamics and childbirth experiences in rural and urban Peru: a qualitative study
Source: BMC Pregnancy Childbirth. 2021 Feb 15;21:135. doi: 10.1186/s12884-021-03586-y (PMC7885576; doi:10.1186/s12884-021-03586-y)
Supplement: Supplementary file 3 — Additional file 3: Supplementary File 3. Provider’s Interview Guide. [file 12884_2021_3586_MOESM3_ESM.pdf]

Interview Guide (Healthcare Providers)

Introduction:
"Thank you for taking the time to talk to me today. The purpose of this interview is to listen to providers regarding their experiences with childbirth care. There are no right or wrong answers to these questions, I would like to hear your honest and personal opinions on these topics. We look forward to using this learning to better understand these collective experiences and use these ideas to make suggestions for improvements in childbirth both at home and in the clinic. The interview is expected to take 45 minutes to an hour, depending on your answers. If you have any questions during our conversation, feel free to ask me. I would like to make it clear that you do not have to answer a question if the question makes you uncomfortable and you can pause or stop the interview at any time. Our whole conversation will be recorded in audio, but everything we talk will remain anonymous. Your name and information will not be connected to your answers.
If you are ready, I can start recording?"

Interview Questions:

1. How did you become interested and involved in this job?

Patient Trust

2. What are some ways you can establish trust with your patients?
   - -Have these methods changed during your career?
   - -What factors influenced a change in these methods?
   - -Do some methods to establish trust seem to work better than others?

3. Have you found that establishing trust changes the experience for you and/or your patient?
   - -In your opinion, how does this trust impact childbirth practices and results?
   - -Is it important? Why?

4. Do you feel that taking the time to establish these relationships with your patients is achievable?
   - -What are some of the barriers to establish trust with your patients?
   - -How do you deal with these barriers?

Facilitators and Barriers to Care

5. How does your workplace affect your practice?
   - -What are some of the advantages of providing care in a clinic/home?
   - -What are some of the disadvantages of providing care in a clinic/home?

6. Do you feel that you have the necessary resources in your workplace?
   - -What kind of resources do you need?
   - -What are the barriers to obtaining or using these resources?

7. Who is typically present during childbirth?
   - -Are the patient's relatives sometimes present?
   - -Are other birth attendants sometimes present?
   - -Does anyone help you? Who?
   - -How do others help you?

Respectful Care

8. What kind of complications have your patients experienced?
   - What happened?
   - How did you know they were complications?
   - How did you act?

9. During care, have your patients ever expressed that they were not satisfied with the care of childbirth?
   - What did they say or do?
   - How did you deal with your patients' distress or negative experience?
   - What was the result of your actions?

10. Do you feel that it is generally easy or difficult to keep your patient informed at all times and maintain communication with them?
   - What factors contribute to the quality of this communication?
   - How do you deal with barriers?

Other Methods of Care

11. What are your opinions on how other midwives or traditional birth attendants provide birth care?
   - What experiences have shaped this view?
   - Have you worked directly with other attendees?
   - How are their experiences different from yours?

12. Have any of your patients expressed that their care differs from other care they have received? Tell me more about this.
   - How did they express this to you?
   - What were some of the differences?
   - What are your views on these differences?

After Childbirth

13. What typically happens immediately after a child is born?
   - Do you direct your attention to the baby?
   - Who attends to the mother and who cares for the baby?
   - In your experiences, do you feel like you give the mother and baby everything they need?
   - What are some of the things you provide?

14. Tell me about the most memorable negative childbirth experience and/or result you have ever attended. How was this experience different from the most memorable positive birth experience and/or result?
   - What factors contributed to either of the results?
   - Did the mother and/or baby need emergency care? Tell me about these experiences.
   - Did the mother and/or baby survive?
   - What made these two particular experiences memorable?

15.Before we finish our conversation, is there anything else you want to add regarding your experiences?

Conclusion:
"Thank you for taking the time to speak to me today, I appreciate your responses. Do you have any questions? If not, I'll turn off the recording now."

UCSF Institute for Global Health Sciences
